# Supplementary material for: Fabrication of Double Emission Enhancement Fluorescent Nanoparticles with Combined PET and AIEE Effects
Source: Molecules. 2020 Dec 4;25(23):5732. doi: 10.3390/molecules25235732 (PMC7731327; doi:10.3390/molecules25235732)
Supplement: Supplementary file 1 [file molecules-25-05732-s001.pdf]

Supporting Information

Fabrication of double emission enhancement fluorescent nanoparticles with combining PET and AIEE effects

Hsing-Ju Wu, Cheng-Chung Chang *

1. General procedure for the synthesis of probes

Cysteine probes (naphthalimide derivatives, CS)-synthesis of the naphthalimide derivative is shown in steps (i), (ii), (iii) of **Figure S1**. The first stage of the reaction, in which commercial starting material 4-bromo-1,8-naphthalic anhydride was reacted with the N-butylamine, was performed conveniently in ethanol under room temperature. Next, the samples were subjected to the Suzuki and Heck coupling reaction, respectively.

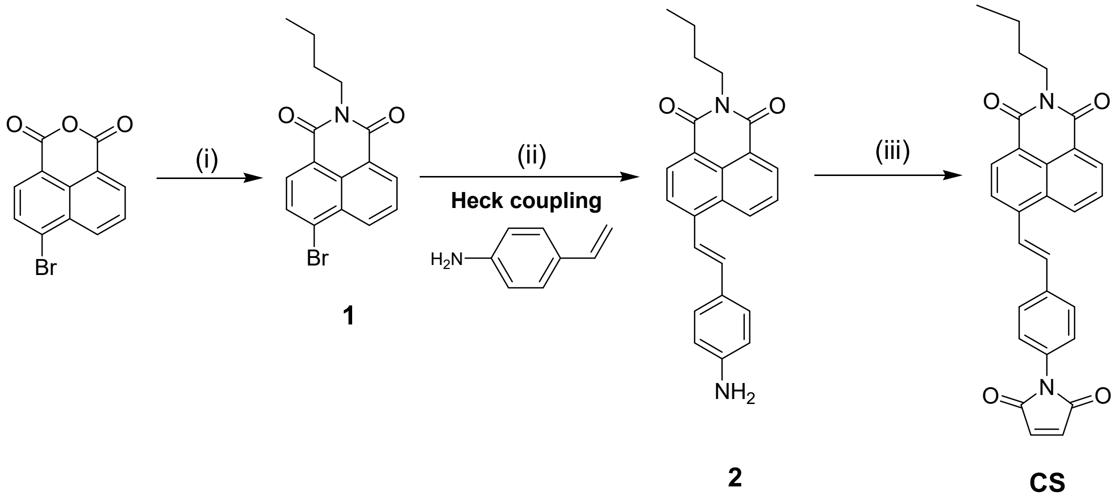

Figure S1: Reagents and conditions: (i) N-butylamine, EtOH, r.t. 24 h; (ii) $Pd(OAc)_2$ /(*o*-tol)$_3$P, 4-aminostyrene, MeCN/Et_3N, N_2, reflux 48 h; (iii) (a) maleic anhydride, $CHCl_3$, reflux 24 h, (b) sodium acetate, acetic anhydride, 90-100 °C 24 h; (iv) pinacolborane, Pd(PPh)$_3$Cl$_2$, dioxane/Et_3N, N_2, reflux 12 h; (v) K_2CO_3, $Pd(OAc)_2$/(o-tol)$_3$P, compound **3**, DME/H_2O, N_2, reflux 48 h.

*1.1. N-butyl-4-bromo-1,8-naphthalimide (compound **1**):*

4-Bromo-1, 8-naphthalic anhydride (2.77g, 10 mmole) and N-butylamine (0.88g, 12 mmole) was stirred in ethanol solution (20 mL) under room temperature for 12h. The system was filtered to remove the excess amine and the collected precipitate was crystallized from acetone/ethanol to get 2.6 g of white solid (yield: 78%). Data for compound **1**: ^{1}HNMR (400Hz, $CDCl_3$, δ in ppm)= 8.65 (d, J = 8.0 Hz, 1H), 8.56 (d, J = 8.2 Hz, 1H), 8.41 (d, J = 8.0 Hz, 1H), 8.03 (d, J = 8.0 Hz, 1H), 7.85 (dd, J = 8.0,8.2 Hz, 1H), 4.17 (t, J = 8 Hz, 2H), 1.69 (m, 2H), 1.44 (m, 2H), 0.97 (t, J = 8 Hz, 3H).

*1.2. N-butyl-4-(4-aminostyryl)-1,8-naphthalimide (compound **2**):*

Compound **1** (1.66g 5 mmole) was added into to a high-pressure bottle containing a mixture of palladium (II) acetate (8 mg) and tri-o-tolyl phosphine (80 mg), then to which was added the solvent pair (triethylamine 5 ml / acetonitrile 15 ml) and 4-vinylaniline (7 mmole). The bottle was then sealed after bubbling with nitrogen for 10 min. After keeping the system under ~105°C for 48 h, the system was cooled to room temperature and then extracted with CH_2Cl_2 / H_2O twice. The organic layer was then dried by with $MgSO_4$ and evaporated in vacuum. The residue was subjected to chromatography on a silica gel by using acetone / hexane (1/3, Rf = 0.4). The 1.3g of red solid was then obtained by recrystallizing with acetone / hexane (yield: 72%). Data for compound **2**: ^{1}H NMR （400 Hz, DMSO-d_6）： δ = 8.96 (d, J = 8.0 Hz, 1H), 8.50 (d, J = 8.2 Hz, 1H), 8.41 (d, J = 8.0 Hz, 1H), 8.15 (d, J = 8.2 Hz, 1H), 7.85 (t, J = 8 Hz, 1H), 7.85 (d, J = 16.0 Hz, 1H), 7.55 (d, J = 7.8 Hz, 2H), 7.46 (d, J = 16.0 Hz, 1H), 6.61 (d, J = 7.8 Hz, 2H), 5.61 (s, 2H), 4.03 (t, J = 8 Hz, 2H), 1.60 (m, 2H), 1.34 (m, 2H), 0.91 (t, J = 8 Hz, 3H) ppm.

*1.3. N-butyl-4-(4-maleimidostyryl)-1,8-naphthalimide (**CS**)*

Compound **2** (0.74g, 2 mmol) and maleic anhydride (0.6g, 6 mmol) were placed in a dry round bottom flask. Chloroform (20 mL) was added and the solution was heated to reflux for 24 h. The mixture was then filtered and the recovered solid was rinsed liberally with chloroform and then dried under vacuum. To this solid was added acetic anhydride (100 mL) and sodium acetate (2 mmol) and the reaction was refluxed for another 90 min. The mixture was cooled and the solution was washed with water, dried over $MgSO_4$, filtered, and evaporated. The compound was purified by silica gel column chromatography (acetone / hexane, 1/3) to give 0.5 g of yellowish solid (yield: 55%). Data for compound **CS**: ^{1}H NMR (400MHz, $CDCl_3$, δ in ppm): δ = 8.65 (d, J = 8.0 Hz, 1H), 8.61 (d, J = 8.2 Hz, 1H), 8.57 (d, J = 8.0 Hz, 1H), 8.01 (d, J = 8.2 Hz, 1H), 7.91 (d, J = 16.0 Hz, 1H), 7.81 (t, J = 8.0 Hz, 1H), 7.74 (d, J = 7.8 Hz, 2H), 7.46 (d, J = 7.8 Hz, 2H), 7.35 (d, J = 16.0 Hz, 1H), 6.90 (s, 2H), 4.20 (t, J = 8 Hz, 2H), 1.74 (m, 2H), 1.46 (m, 2H), 0.99 (t, J = 8 Hz, 3H). HRMS (ESI, *m/z*): $[M+H]^+$ 451.16; found, 450.92. Anal. Calcd for $C_{28}H_{22}N_2O_4$: C, 74.65, H, 4.92; N, 6.22; found: C, 73.95; H, 4.95; N, 6.11.

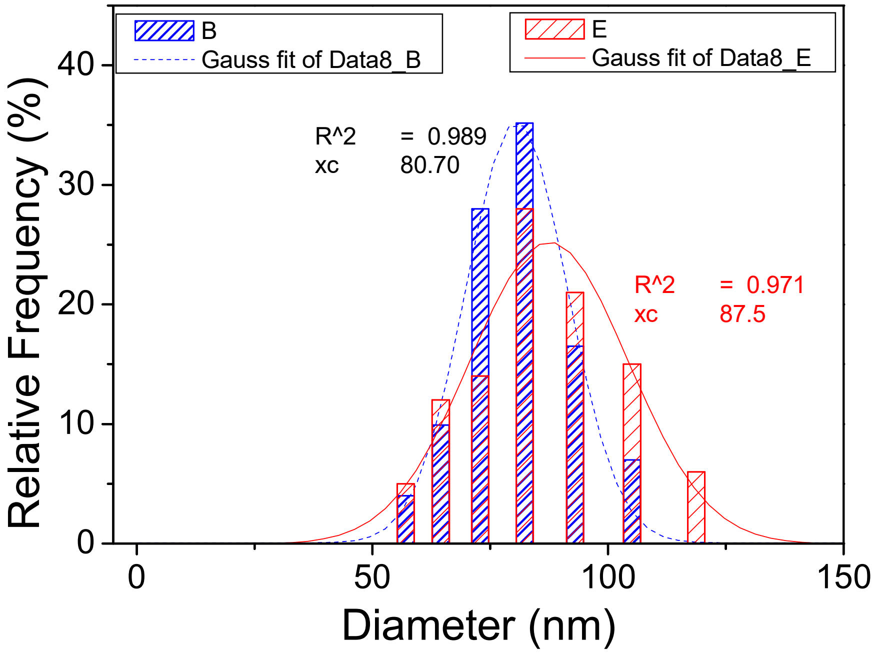

Figure S2. Dynamic light scattering (DLS) data for CSP-NP@OH nanpparticles (blue) and CSP-NP@OH/NH_2 (red).

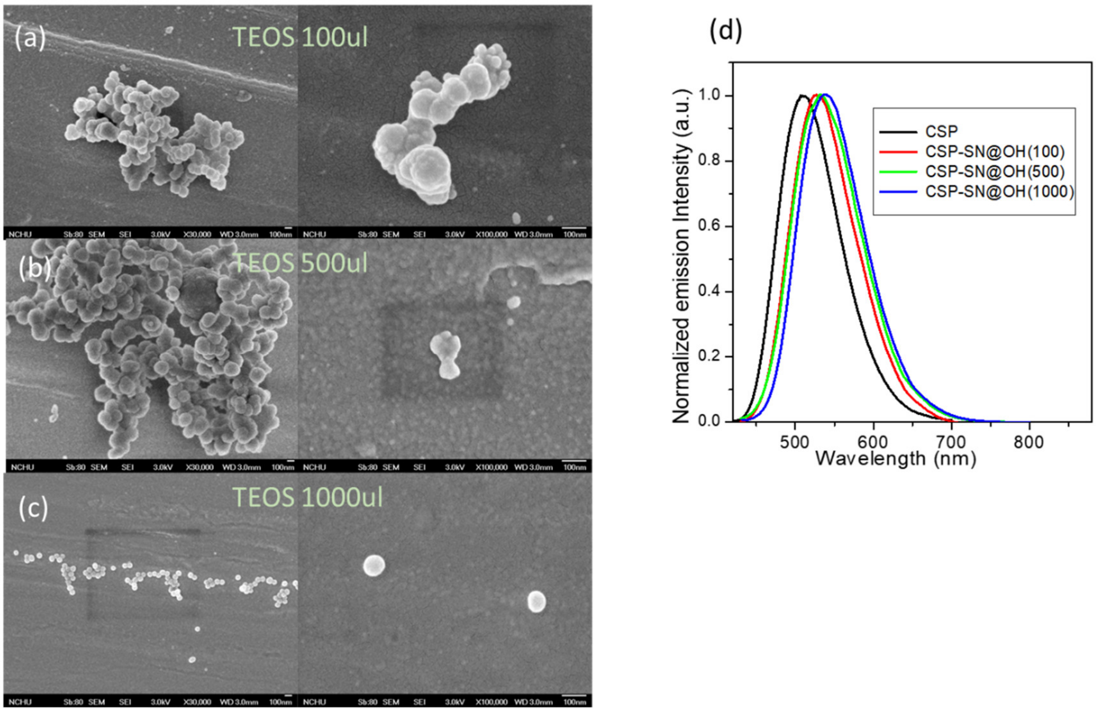

Figure S3. SEM images of preparation CSP-SN@OH from (a) 100; (b) 500; (c) 1000 uL of TEOS. (d) their response emission spectra in EtOH, with respect to CSP monomer.

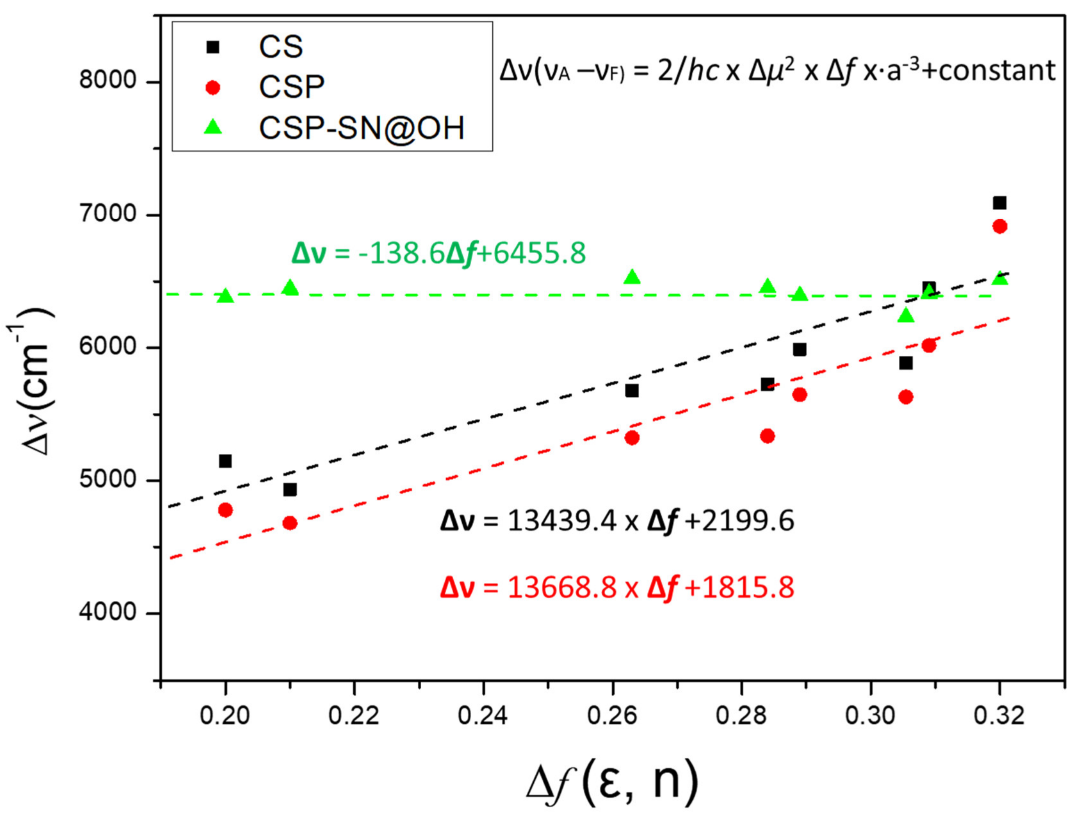

Figure S4. The slopes of the Lippert (Δν, Stokes shift vs Δ*f,* orientation polarizability) plots from solvent effect results of CS, CSP and CSP-SN@OH, which reflect dipole moment exchange Δ***μ*** (dot line).

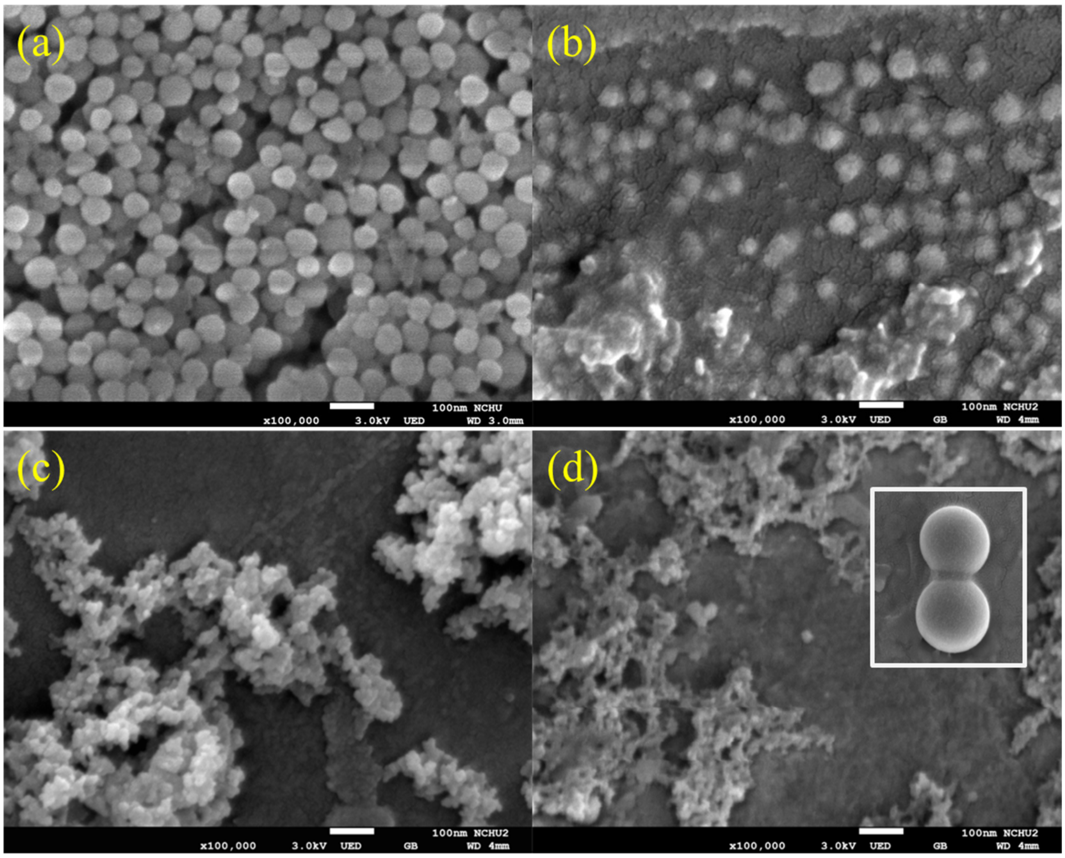

Figure S5. SEM images of preparation CSP-SN@OH/NH_2 from TEOS/APTMS= (a) 8/2; (b) 7/3; (c) 6/4; (d) 5/5 for 1000 μL total volume.

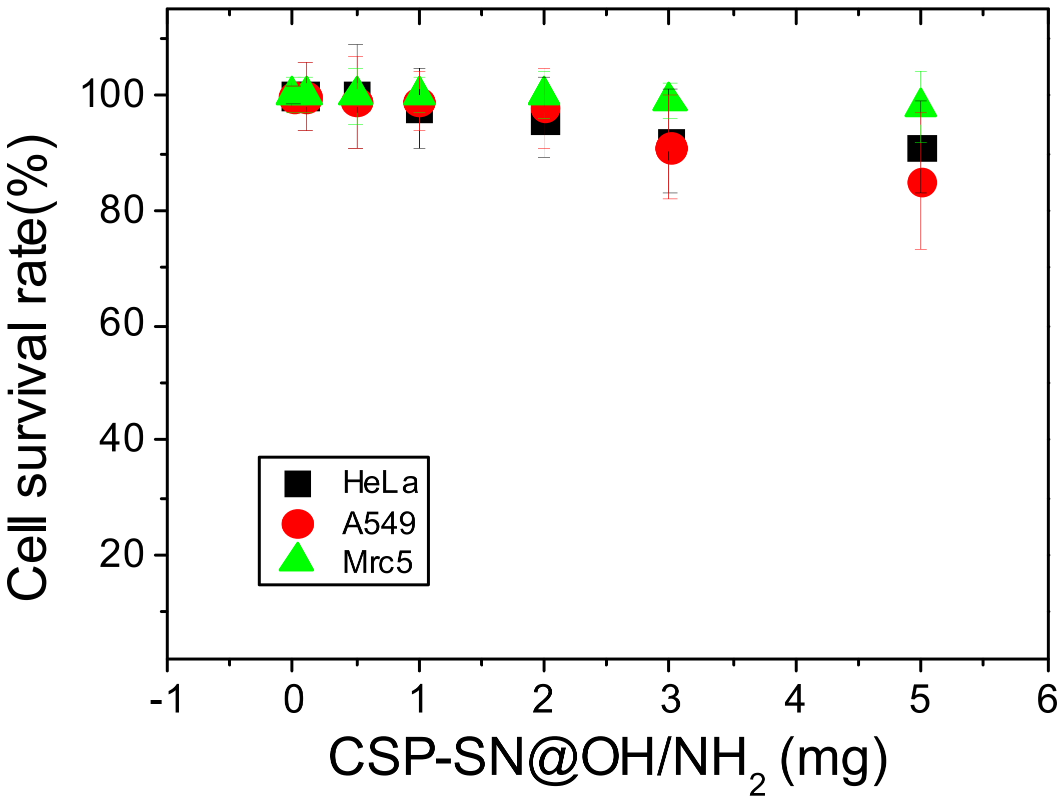

Figure S6. The cytotoxicity evaluation of CSP-SN@OH/NH_2 in HeLa (human cervical carcinoma), A549 (human lung adenocarcinoma cancer), and MRC-5(human normal lung fibroblast) cells.

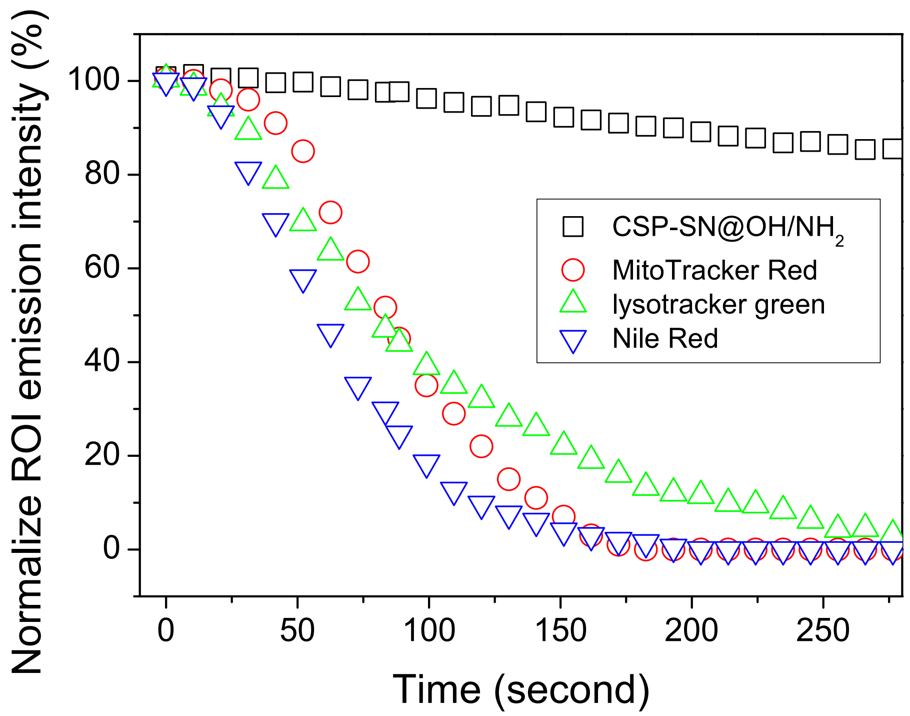

Figure S7. The performance of CSP-SN@OH/NH_2 for long-term imaging was benchmarked against that of MitoTracker™ Red (mitochondria tracker) (excitation lase 532 collected filter wave length: 560 nm long pass), lysotracker green (lysosome tracker) (excitation lase 488 collected filter wave length: 510 nm long pass) and Nile Red (Lipid droplet tracker) (excitation lase 532 collected filter wave length: 560 nm long pass) directly in A549 living cells over time. CSP-SN@OH/NH_2 adopted the same condition as Figure 5e.
